# Real-World Outcomes in Patients with Branch Retinal Vein Occlusion- (BRVO-) Related Macular Edema Treated with Anti-VEGF Injections Alone versus Anti-VEGF Injections Combined with Focal Laser

**DOI:** 10.1155/2021/6641008

**Published:** 2021-05-19

**Authors:** Meredith E. Thomley, Cole N. Gross, Ana Preda-Naumescu, Kelly S. Chen, Thomas Swain, John O. Mason III, Jason N. Crosson

**Affiliations:** ^1^The University of Alabama, Birmingham School of Medicine, Birmingham, AL 35233, USA; ^2^Retina Consultants of Alabama, The University of Alabama, Birmingham School of Medicine, Birmingham, AL 35233, USA; ^3^Ophthalmology Resident at the Callahan Eye Center, University of Alabama, Birmingham Department of Ophthalmology, Birmingham, AL 35233, USA; ^4^Department of Ophthalmology and Visual Sciences, Birmingham, AL 35233, USA; ^5^Retina Consultants of Alabama, The University of Alabama, Birmingham Department of Ophthalmology, Birmingham, AL 35233, USA

## Abstract

The purpose of this study was to assess outcomes in a real-world nonclinical trial setting of antivascular endothelial growth factor (VEGF) injections alone vs. focal laser combined with anti-VEGF injections in patients with branch retinal vein occlusion- (BRVO-) related macular edema (ME). This study included 88 BRVO with ME patients who were treated over three years at both a tertiary referral center in the Birmingham metropolitan area and satellites in rural Alabama. One group received only anti-VEGF injections (*n* = 56); the other group received both anti-VEGF injections and focal laser (*n* = 32). The following outcome measures were evaluated: initial and final visual acuities (VA), initial central subfield thickness (CST) on OCT, number of injections, number of lasers, percentage of patients with a gain of 3 lines of VA, percentage of patients with VA better than or equal to 20/40, and percentage of patients with VA worse than or equal to 20/200. We found that there was no difference in initial VA (*p*=0.913) or CST (*p*=0.961) between the two groups. The injection only group required a median of 7 injections, while the combination group required a median of 4 injections, but this was not a statistically significant difference (*p*=0.117). There was no difference in final VA (*p*=0.414) or any of the other visual outcomes between the two groups. In conclusion, focal laser did not decrease the number of injections required or improve the VA in BRVO-related ME. Although visual outcomes were similar in both groups, focal laser does not appear to be of additional benefit in BRVO-related ME in the anti-VEGF era.

## 1. Introduction

Branch retinal vein occlusion (BRVO) is the second most frequent major retinal vascular disease. There are approximately 144,000 new cases of BRVO in the United States each year [1–3]. The most common reason for decreased vision secondary to BRVO is macular edema (ME) [4]. Allowing macular edema to go untreated may result in profound and potentially irreversible vision loss. For macular edema secondary to BRVO, focal laser photocoagulation has historically been the mainstay for management and has been used with varied success [5]. Concurrent with the development of optical coherence tomography (OCT) and its rise to a vital role in any retina clinic, anti-VEGF injections have become the first-line treatment for patients with macular edema secondary to BRVO as well as many other retinal diseases [6]. Anti-VEGF injections have shown great efficacy but not without limitations and potential adverse effects. There is always the potential concern for endophthalmitis, and often, there is a need for prolonged injection courses leading to the need for frequent office visits for injections, which can be quite a burden for patients that often have other medical comorbidities. Furthermore, there is a subset of patients that have a minimal response to these injections [7]. Intravitreal corticosteroids (such as intravitreal triamcinolone and dexamethasone) are also an effective treatment for ME secondary to BRVO (and many other retinal diseases) that may offer the benefit of less frequent injections, but at the expense of an increased risk of cataracts and glaucoma [8–10]. In addition, combination therapy utilizing anti-VEGF injections, corticosteroids, and/or laser can be employed as can vitrectomy surgery (when vitreomacular traction is contributing to the BRVO-related macular edema).

Numerous small studies have suggested that focal laser combined with anti-VEGF injections may reduce the number of injections needed and may offer superior visual outcomes to anti-VEGF alone [11–13]. This, however, is controversial, and a small (32 patients) recent prospective randomized study presented evidence to the contrary [14]. Certainly, if focal laser can be shown to benefit patients visually or at least limit the number of injections in the long run, this could reduce the treatment burden for many patients. Conversely, it will also be useful to know if focal laser is not beneficial in the VEGF era, as it would be best to not subject patients to photocoagulation of retinal tissue and the risk of laser scarring/creep if there is really no benefit [15]. Herein, we assess our real-world outcomes in BRVO patients with ME treated with injections alone versus injections plus focal laser with the aim of further clarifying the treatment of BRVO-related ME.

## 2. Materials and Methods

Institutional review board (IRB) approval (protocol number X070802011) was obtained prior to collection of patient data from the University of Alabama-Birmingham IRB. The study conducted is HIPAA compliant and adhered to the tenets of the Declaration of Helsinki.

This study was a retrospective chart review of BRVO patients with ME diagnosed and treated within the last 3 years. Study participants were identified by chart review of patients of retina specialists at Retina Consultants of Alabama at Callahan Eye Hospital in Birmingham, Alabama. Study participants included 88 eyes from 88 patients (mean age: 72.2 ± 9.9 years); 56 participants received only intravitreal anti-VEGF injections (63.6% of patients), and 32 received both intravitreal anti-VEGF injections as well as focal laser (36.4%). Injections in both groups were primarily bevacizumab, though a minority of eyes in both groups that did not respond to bevacizumab received ranibizumab or aflibercept. As this was a retrospective review, the decision to move forward with injections and/or laser for each patient was made by the treating physician at the time of treatment. Subjects included patients from both the Birmingham metropolitan area and satellites in more rural areas of Alabama. Patients were excluded if they were not treatment naïve, if they had less than 5 months of follow up, if they were less than 18 years old, or if they had other etiologies such as diabetic retinopathy that may cause ME or contribute to a less than expected visual potential/recovery.

The following outcome measures were evaluated and compared in both groups: initial and final visual acuities (VA), initial and final central subfield thickness (CST) on OCT, number of injections, number of lasers, percentage of patients with a gain of 3 lines of VA, percentage of patients with VA better than or equal to 20/40, and percentage of patients with VA worse than or equal to 20/200 and median change in VA. Though many different OCT platforms exist (spectral domain OCT, enhanced depth imaging, and swept source OCT), spectral domain OCT was used in all patients in this study. VA was measured on a standard Snellen eye chart with best spectacle correction in place.

Data abstracted were summarized and analyzed. The median and interquartile range were used to describe continuous data since they were not normally distributed, except age. Continuous variables were compared using the *t*-test or Wilcoxon rank sums test (nonparametric *t*-test) as appropriate. Categorical comparisons were made using Fisher's exact test, and the number of injections was compared using Poisson regression. The level of significance was set to <0.05. All analyses were conducted in SAS version 9.4 (SAS Institute, Cary, NC).

## 3. Results and Discussion

### 3.1. Results

There were a total of 88 patients in this retrospective study (mean age: 72.2 ± 9.9 years). 56 patients received only intravitreal anti-VEGF injections (63.6%) and 32 received both intravitreal anti-VEGF injections and focal laser (36.4%). There were no complications in either group. There was no difference in initial VA (*p*=0.913). Groups were not found to have a statistically significant difference in presenting central subfield thickness on OCT (median of 456 *μ*m vs. 487 *μ*m, *p*=0.961) (Table 1).

**Figure 1:**
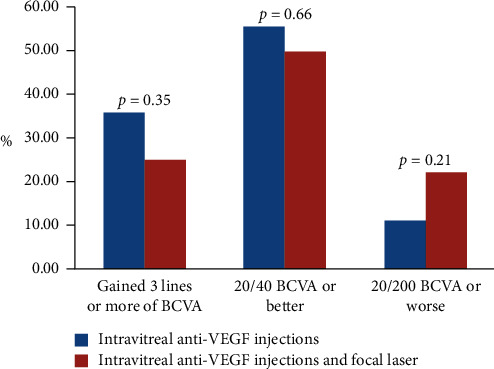
Visual acuity outcome comparison between anti-VEGF injections alone and anti-VEGF injections combined with focal laser.

In regard to visual acuity outcomes, there was no statistically significant difference between the groups. There was no difference in final VA, the percentage of patients with 20/40 or better final VA, or the percentage of patients with 20/200 or worse final VA (*p*=0.414, *p*=0.662, and *p*=0.213, respectively). The percentage of patients that gained 3 or more lines was 35.7% in the injection only group and 25% in the combination group, and this was not statistically significant (*p*=0.348). The injection only group gained a median of 2 lines, while the combination group gained a median of 2.5 lines, and this was not statistically significant (*p*=0.833) (Table 2, Figure 1).

**Figure 2:**
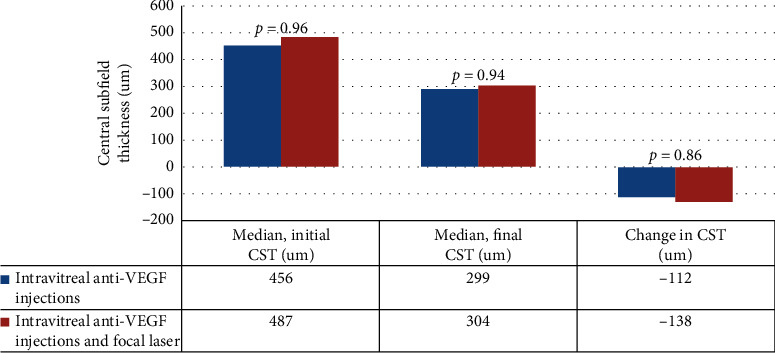
Central subfield thickness on OCT comparison between anti-VEGF injections alone and anti-VEGF injections combined with focal laser.

In addition, there was no statistical significant difference found between the numbers of injections each group received. The injection only group required a median of 7 injections (interquartile range: 4.5–9.5), while the combination group required a median of 4 injections (interquartile range: 3.0–7.5) (*p*=0.117). At first look, this difference appears significant but is accounted for by the large difference in interquartile range (Figure 2).

**Figure 3:**
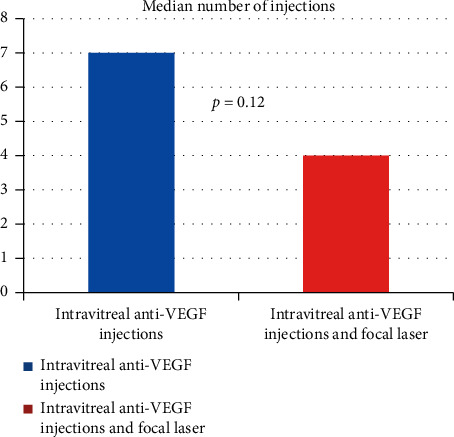
Number of intravitreal injections comparison between anti-VEGF injections alone and anti-VEGF injections combined with focal laser.

There was no statistical difference in final (median of 299 *μ*m vs. 304 *μ*m, *P* value of 0.944) or change (median of −112 *μ*m vs. −138 *μ*m, *P* value of 0.860) in central subfield thickness (Figure 3).

### 3.2. Discussion

BRVO with associated ME is a common and potentially vision-threatening diagnosis. Well-validated randomized studies have shown the efficacy of both anti-VEGF intravitreal injections and focal laser in the treatment of BRVO macular edema [13, 14]. Intravitreal injection of anti-VEGF have now become the most common means of treating this condition, but the use of focal laser is still widely performed, often as an adjunctive therapy. However, there have been a limited number of studies that have shown a significant benefit in terms of improved visual acuity and/or decreased number of intravitreal injections when focal laser is used as an adjunctive therapy [7, 11, 12].

From a provider and patient perspective, goals for combination treatment are intuitive: reduction of ME and greater improvement in visual acuity and in a shorter period of time with less treatments. Our study, however, offers data to suggest that focal laser may not be beneficial in the anti-VEGF era. We did not see any difference in visual outcome between groups. We also did not find any statistically significant reduction in the number of injections between groups. We considered the fact that, in our retrospective study, patients with worse BRVOs, worse ME, and more macular ischemia may have been patients more likely to have been treated with combination treatment (injections plus focal laser). One could hypothesize that this is why the combination group did not have better vision or receive less injections. However, both groups had similar initial visual acuity and CST on OCT, suggesting that the vein occlusions were of similar severity in both groups. Also, visual outcomes were not worse, and patients did not receive more injections in the combination group. This suggests that combination treatment still results in similar visual outcomes compared to injections alone. Furthermore, it suggests that the BRVOs in the combination group did not likely have more macular ischemia, as one would have expected a worse visual outcome in the combination group if this were the case. Some measures of bias on the part of the treating physician towards doing less injections in patients that have had laser cannot be ruled out, but this seems unlikely since there was no statistically significant difference in the number of injections Still, based on our results, as well as similar small studies in the literature, adding focal laser to the anti-VEGF regiment for BRVO-related ME may not add any additional benefit.

Limitations of our study include its retrospective nature and its relatively small sample size, though our study is larger than many in the literature. In addition, fluorescein angiography and/or OCT angiography (OCTA) were not used to confirm the presence or absence of macular ischemia, though the similar initial and final visual acuities between groups as well as the similar CST on OCT before and after intervention would suggest that neither group had a higher proportion of severely ischemic eyes. The potential biases inherent in a retrospective study design discussed above are also limitations, but the similarities between the initial groups (VA and CST) and the outcomes in our study (no significant difference in the number of injections between the two groups when physicians may be biased to do less injections if laser is performed, similar final VA, and similar final CST) suggest that the two patient groups are comparable and meaningful conclusions can still be made. Finally, the fact that a minority of eyes in each group received aflibercept and ranibizumab makes a strict comparison between bevacizumab and focal + bevacizumab more difficult. Even so, both the injection only and the injection + laser group mostly received bevacizumab, and the use of the other two medications was similar between the two groups. We would also argue that such a comparison is more generalizable to actual real-world clinical practice in which physicians will switch agents if macular edema remains persistent. A unique strength of our study is our diverse patient population, which includes patients from the Birmingham metropolitan area referred to our tertiary referral center and patients from our satellite clinics in rural Alabama. This diverse patient population and the nonclinical trial setting also make our results more generalizable to the real-world setting.

## 4. Conclusion

In conclusion, focal laser combined with anti-VEGF injections was not shown to be of benefit when compared to anti-VEGF injections alone. Our study suggests to clinicians and patients that the addition of focal laser to anti-VEGF injections may not be a useful treatment for BRVO-related macular edema in the anti-VEGF era. The potential adverse effects of focal laser in addition to its financial cost to the patient must be considered when recommending it as an adjunctive treatment. These factors should be discussed prior to its use, so the patient can make a well-informed decision. Rather than adding laser to the anti-VEGF regimen, clinicians may choose to continue with anti-VEGF monotherapy or consider other alternatives such as intravitreal steroids. Further larger prospective studies will be beneficial going forward so as to elucidate the optimal treatment or combination of treatments for patients with BRVO-related ME.

## Figures and Tables

**Table 1:** Demographics in BRVO-related ME: injections only vs. injections plus focal laser.

	Injections only (*N* = 56)	Injections + laser (*N* = 32)	*P* value
Mean age	71.5 ± 10	73.5 ± 9.6	0.36
Presenting VA	75 (50–200)	70 (55–200)	0.91
Initial median IOP	16 (14–17)	16 (13.5–17)	0.82
Initial central subfield thickness in *μ*m—OCT, median (interquartile range)	456 (389–567)	487.5 (330.5–627.5)	0.96

**Table 2:** Outcomes in BRVO-related ME: injections only vs. injections plus focal laser.

	Injections only	Injections + laser	*P* value
Gained 3 or more lines on letter chart, *N* (%)			0.35
Yes	20 (35.7)	8 (25)	
No	36 (64.3)	24 (75)	
Lines of improvement, median (interquartile range)	2 (1–4)	2.5 (1–3.5)	0.83
% of patients with 20/40 VA or less	55.36	50.0	0.66
% of patients with 20/200 VA or more	10.71	21.88	0.21
# of injections, median (interquartile range)	7.0 (4.5–9.5)	4.0 (3.0–7.5)	0.12
Final central subfield thickness in *μ*m—OCT, median (interquartile range)	299 (255–379)	304 (282–373)	0.944

## Data Availability

The data used to support the findings of this study are available from the corresponding author upon request.
